# Neural Correlates of the Severity of Cocaine, Heroin, Alcohol, MDMA and Cannabis Use in Polysubstance Abusers: A Resting-PET Brain Metabolism Study

**DOI:** 10.1371/journal.pone.0039830

**Published:** 2012-06-29

**Authors:** Laura Moreno-López, Emmanuel A. Stamatakis, Maria José Fernández-Serrano, Manuel Gómez-Río, Antonio Rodríguez-Fernández, Miguel Pérez-García, Antonio Verdejo-García

**Affiliations:** 1 Department of Personality, Evaluation and Psychological Treatment, University of Granada, Granada, Spain; 2 Division of Anaesthesia, School of Clinical Medicine, University of Cambridge, Cambridge, United Kingdom; 3 Department of Psychology, University of Jaén, Jaén, Spain; 4 Service of Nuclear Medicine of the Hospital Virgen de las Nieves of Granada, Granada, Spain; 5 Centro de Investigación Biomédica en Red de Salud Mental, CIBERSAM, University of Granada, Granada, Spain; 6 Institute of Neurosciences Federico Olóriz, University of Granada, Granada, Spain; University of Manchester, United Kingdom

## Abstract

**Introduction:**

Functional imaging studies of addiction following protracted abstinence have not been systematically conducted to look at the associations between severity of use of different drugs and brain dysfunction. Findings from such studies may be relevant to implement specific interventions for treatment. The aim of this study was to examine the association between resting-state regional brain metabolism (measured with 18F-fluorodeoxyglucose Positron Emission Tomography (FDG-PET) and the severity of use of cocaine, heroin, alcohol, MDMA and cannabis in a sample of polysubstance users with prolonged abstinence from all drugs used.

**Methods:**

Our sample consisted of 49 polysubstance users enrolled in residential treatment. We conducted correlation analyses between estimates of use of cocaine, heroin, alcohol, MDMA and cannabis and brain metabolism (BM) (using Statistical Parametric Mapping voxel-based (VB) whole-brain analyses). In all correlation analyses conducted for each of the drugs we controlled for the co-abuse of the other drugs used.

**Results:**

The analysis showed significant negative correlations between severity of heroin, alcohol, MDMA and cannabis use and BM in the dorsolateral prefrontal cortex (DLPFC) and temporal cortex. Alcohol use was further associated with lower metabolism in frontal premotor cortex and putamen, and stimulants use with parietal cortex.

**Conclusions:**

Duration of use of different drugs negatively correlated with overlapping regions in the DLPFC, whereas severity of cocaine, heroin and alcohol use selectively impact parietal, temporal, and frontal-premotor/basal ganglia regions respectively. The knowledge of these associations could be useful in the clinical practice since different brain alterations have been associated with different patterns of execution that may affect the rehabilitation of these patients.

## Introduction

Drug addiction has been associated with neuroadaptations in brain systems involved in motivation, memory and executive control [1]. Functional neuroimaging studies have revealed that the use of different classes of drugs is associated with dysfunctions in a range of overlapping brain regions including ventromedial and dorsolateral prefrontal cortex (DLPFC), anterior cingulate cortex, inferior frontal gyrus, insula, amygdala, basal ganglia and cerebellum [2]. These dysfunctions may explain the overlapping cognitive deficits observed in users of different drugs, including working memory, inhibitory control, flexibility or decision-making deficits [3]. In addition, some specific effects have been described for the association between cocaine use and the parietal cortex [4], [5], [6], heroin use and the temporal cortex [7], MDMA use and occipital, hippocampus and thalamic regions [8], [9], and cannabis use and the premotor cortex [10], [11]. These associations are congruent with relatively specific neuropsychological deficits pertaining to attention and cognitive control in cocaine users, long-term memory in opiate users, visuospatial memory in MDMA users, and psychomotor function in cannabis users [3], [7]. In spite of the available evidence, in vivo studies have not been systematically conducted to look at associations between severity of use of different drugs and brain dysfunction. In fact, several neuroimaging studies have failed to detect such link or have provided controversial results (reviews in [12]). These negative findings and controversies might be linked to the impact of relevant confounding variables. One of these is the fact that neuroimaging studies have frequently tested drug users currently using drugs or having brief periods of abstinence (24–48 h) [13], [14]; under these conditions, the presumed link between lifetime drug use and brain dysfunction could be masked by several other factors, including recent drug use or psychological symptoms associated with withdrawal and short-term abstinence [2]. Another key variable for consideration is the concurrent use of multiple types of substances, which can introduce significant confounds in the interpretation of the data.

The current study aimed to specifically address the association between lifetime estimates of use of different types of drugs and brain functioning (as measured by 18F-fluorodeoxyglucose Positron Emission Tomography (FDG-PET), statistically controlling for the concurrent use of other drugs in a sample of polysubstance users with prolonged abstinence from all drugs. Specifically, we examined the link between lifetime estimates of amount and duration of use of cocaine, heroin, alcohol, MDMA and cannabis and brain metabolism (BM). Based on previous findings we hypothesized that exposure to all substances would be significantly associated with overlapping reductions of metabolism in frontal, limbic, striatal and cerebellar regions, whereas cocaine and heroin use would specifically correlate with parietal and temporal cortices respectively.

## Methods

### 1. Participants

Forty-nine substance dependent individuals (SDI), forty-one men and eight women with a mean age of 32.67 years, were recruited during their stay in an inpatient treatment program at the centre “Proyecto Hombre” in Granada (Spain). This centre provides a controlled setting for the treatment of substance use disorders. Selection criteria for participants in this study were (i) meeting the DSM-IV criteria for substance dependence; (ii) absence of documented comorbid mood or personality disorders, as assessed by clinical reports; (iii) absence of documented head injury or neurological disorders; and (iv) to have minimum abstinence duration from all drugs consumed (except for tobacco) of 15 days before testing. In fact, SDI participants had overall much longer abstinence duration (mean of 32.94, SD = 11.25 weeks); for that reason it was possible to rule out the presence of withdrawal symptoms or brain function alterations associated with the acute or short term effects of the drugs. None of the participants were experiencing withdrawal symptoms as assessed by routine medical examination or were enrolled in opioid substitution treatments with methadone or other pharmacological treatments (e.g., benzodiazepines) during the course of the assessment interview/PET evaluations.

### 2. Testing Protocols and Procedures

This study was approved by the Human Subjects Committee of the University of Granada. One-step Syva urine drug screens for amphetamines, benzodiazepines, cannabis, cocaine and opiates were conducted to confirm abstinence at time of testing. Participants were evaluated individually in two different sessions. During the first session, the participants were provided with information about the study and were encouraged to ask questions to ascertain they fully understood the rational for the study and were clear on the details for participation. The participants then provided written consent and were assessed with the Interview for Research on Addictive Behaviours [15] in the “Proyecto Hombre” installations”. During the second session, each patient was accompanied by a therapist to the nuclear medicine service of the “Virgen de las Nieves” hospital, where the PET neuroimaging session took place. This second session usually took place one week after the first session was completed.

### 3 Tools

#### 3.1 Drug use information

The *Interview for Research on Addictive Behaviour* [15] was used to examine the severity of drug use. This interview evaluates, by means of a brief interview, the quantity (average dosing), frequency (number of drug taking episodes per month), and duration (years of duration) of the use of different substances that can produce physical or psychological dependence, including cocaine, heroin, alcohol, MDMA and cannabis which were the main drugs of choice in the present study. For every substance the participant had actually used, the following information was requested: (i) the average dose of each target drug taken in each episode of use (number of grams for cocaine and heroin, number of units for alcohol, considering that 25 cl. of hard liquor (e.g., scotch) equals 2 units, while 25 cl. of wine or beer equals 1 unit, number of pills for MDMA and number of cigarettes for cannabis); (ii) the frequency of these consumption episodes per month (daily, between one and three times upon a week, once a week, between one and three times upon a month, or once a month); and (iii) the number of years that elapsed since the onset of use. From this information, we obtained two indices for each of the drugs used: (i) *Amount* (average dose x frequency), an index of monthly use of the drug; and (ii) *Duration* of drug use, measuring number of years of exposure to the drug. The main sociodemographic and clinical features from the sample are displayed in Table 1.

**Table 1:** Sociodemographic data and clinical features of the participants.

Subjects Characteristics				
*Gender*				
Male	41			
Female	8			
	*Mean*	*S.D.*	*Maximum*	*Minimum*
*Age (years)*	32.67	8.03	53	21
*Education (years)*	9.73	2.34	17	5
**Amount of drug use** a				
Substance	*Mean*	*S.D.*	*Maximum*	*Minimum*
Cocaine (g) (46)	48.69	44.83	180	0
Heroin (g) (16)	9.12	20.63	120	0
Alcohol (units) (45)	571.24	489.34	1800	0
MDMA (pills) (23)	13.41	24.31	120	0
Cannabis (cigarettes) (37)	148.57	190.64	750	0
**Duration of drug use (years)**				
*Substance*	*Mean*	*S.D.*	*Maximum*	*Minimum*
Cocaine	7.95	5.95	23	0
Heroin	1.76	3.69	17	0
Alcohol	10.85	7.67	27	0
MDMA	1.40	2.28	8	0
Cannabis	8.31	8.13	29	0
Abstinence (weeks)	32.94	11.25	60	12

a Amount of monthly use of the drug calculated using the average dose (per episode) × frequency (per month).

#### 3.2 PET image acquisition

PET scans were acquired with an ECAT/931 scanner (Siemens CTI ECAT EXACT), at the service of nuclear medicine centre of the hospital “Virgen de las Nieves” in Granada (Spain). For each individual, we obtained 20 minute emission scans 30 minutes after the injection of one dose of 200 MBq of FDG administrated only after levels of glycemia had been checked (they must be below 120 mg/dl) [16]. The subjects were scanned with their eyes open and ears unplugged in a dimly illuminated quiet room. Raw data were processed using an iterative reconstruction technique (OSEM method: 10 iterations, 32 subsets). Images were reoriented in transaxial, coronal and saggital planes. For the analyses reported here the reconstructed images used had 47 axial slices each with an in-plane resolution of 2.57×2.57 and a slice thickness of 3.38 mm.

### 4 Data Analysis

#### 4.1 Preprocessing of PET images

PET images were converted from DICOM to NIFTI format and were spatially normalized to the SPM PET template (Wellcome Department of Cognitive Neurology, London, UK) using linear (12 affine) and non-linear normalization. An average image of all the normalized images was obtained and this acted as a study specific PET template. In this manner we constructed a template in the same modality, from the same scanner as the images we wanted to normalize. The raw PET images were then spatially normalized with linear affine and nonlinear parameters to this study-specific template. Visual inspection revealed this optimized spatial normalization technique produced satisfactory spatial normalization for the PET image of every volunteer.

#### 4.2 Statistical analysis

The spatially normalized PET images were smoothed with an 8 mm^3^ isotropic Gaussian filter and were statistically modeled using the General Linear Model in SPM5. Linear regressions were used to carry out several analyses in order to identify brain areas with a significant correlation between uptake values and measures of the amount and duration of cocaine, heroin, alcohol, MDMA and cannabis use. Not all subjects used all the drugs but since virtually all participants had regularly abused two or more substances, the analyses were conducted on the whole group and results were interpreted in relation to polysubstance use. In each of the analysis, we controlled for the effects of the other substances concurrently used (e.g., analysis of severity of cocaine use controlled for the effects of co-abuse of heroin, alcohol, MDMA and cannabis), age and years of education. We report local maximum peaks that survive a voxel threshold of p<0.001 (uncorrected for multiple comparisons, cluster size ≥100 voxels). We chose to report our findings at this threshold based on numerous similar studies of PET imaging in drug addiction [17], [18].

## Results

The main results are summarized in Table 2.

**Table 2:** Relation between severity of drug use and PET uptake.

Substancee	Voxels-Based Analysis			BA	K_E_	*T*	MNI					
*Amount*												
Cocaine	Parietal Inf L		40		176	3.98	−58		−38		54	
Alcohol	Temporal Mid L		21		267	4.04	−66		−2		−10	
	Frontal Sup L		9		308	3.96	−20		30		36	
	Frontal Sup R		6		365	3.73	22		2		60	
	Frontal Mid R		46		372	3.82	26		22		34	
Cannabis	Frontal Inf Tri L		45		355	4.40	−44		22		30	
	Frontal Mid R		46		125	4.02	24		48		28	
*Duration*												
Heroin	Frontal Mid R	46			876	3.77	44	36		34		
	Temporal Inf R	20			257	4.58	62	−16		−32		
	Temporal Sup R	22			1090	4.19	66	−44		20		
Alcohol	Temporal Inf R	20			238	4.22	64	−16		−30		
	Putamen R				472	3.71	20	−2		8		
	Precentral R	6			274	3.67	50	2		34		
	Fusiform R	20			299	3.65	30	−6		−40		
MDMA	Postcentral L	40			131	3.73	−28	−40		48		
	Frontal Inf Tri R	45			139	3.67	44	34		20		
	Temporal Pole Sup R	20			561	3.67	32	12		−30		

Voxel-based analyses at *p*<0.001 (uncorrected, cluster size >100 voxels). Labels were obtained using the aal toolbox [32]. Broadmann areas were obtained using MRICron (Rorden and Brett, 2000). K_E_ indicate the number of voxels included in the cluster. Stereotaxic coordinates are those listed in SPM5. Inf, inferior; Sup, superior; L, left; R, right; Supp, supplementary; Mid, middle; Orb, orbitalis.

### 1 Amount of Use

Voxel-based analyses showed negative correlations between lifetime amount of cocaine, alcohol and cannabis use and several brain regions. The amount of cocaine used was negatively correlated with a cluster encompassing the left inferior parietal lobule extending to the left postcentral gyrus (Figure 1.1). The amount of alcohol used was negatively associated with three clusters: one cluster included the left middle and superior temporal cortex; a second cluster included the bilateral superior frontal cortex extending to the left DLPFC and the right supplementary motor area; and a third cluster included the right DLPFC extending to the superior frontal gyrus (Figure 1.2). The amount of cannabis use showed a negative correlation with two clusters: one cluster included the left inferior frontal gyrus (pars triangularis) extending to the DLPFC, and a second cluster included the right DLPFC extending to the superior frontal cortex (Figure 1.3). We did not find significant correlations between the amount of heroin or MDMA use and BM measures.

**Figure 1:**
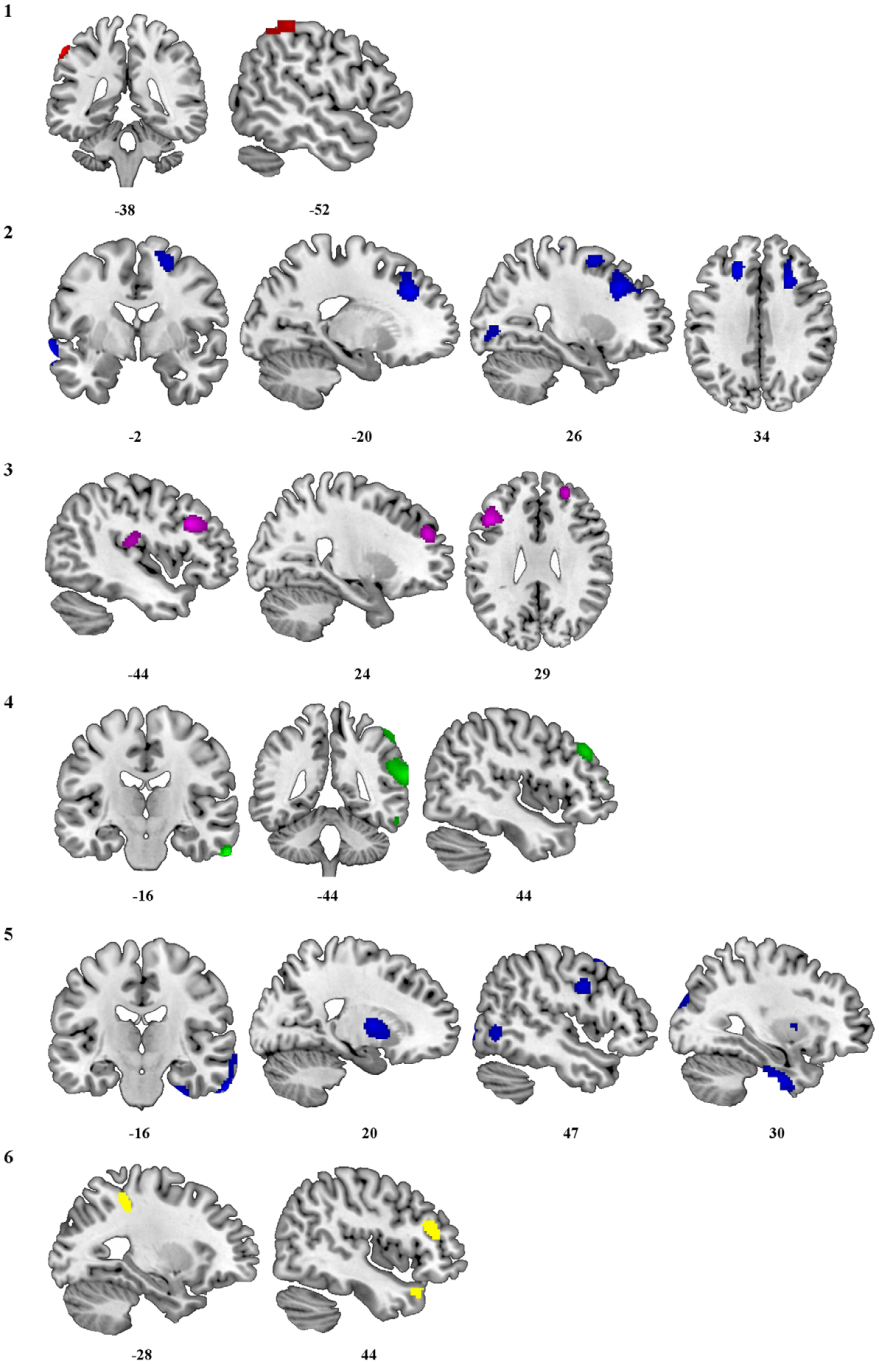
Areas of reduced metabolism predicted by the amount or duration of drug use: (1) amount of cocaine, (2) amount of alcohol, (3) amount of cannabis (4) duration of heroin (5) duration of alcohol and (6) duration of MDMA. Areas of reduced metabolism are superimposed on a T1 weighted MRI image in MRICRON. MNI coordinates are shown underneath each panel.

### 2 Duration of Use

Voxel-based analyses showed negative correlations between duration of heroin, alcohol and MDMA use and several brain regions. Duration of heroin use showed a negative correlation with three clusters, which encompassed the right inferior, middle and superior temporal cortex extending to the right supramarginal and inferior frontal gyrus (Figure 1.4). Duration of alcohol use was negatively correlated with four clusters. One cluster included the right inferior temporal cortex extending to the middle aspect of this area. A second cluster included the right putamen and pallidum. A third cluster encompassed the right precentral and postcentral gyrus and the fourth cluster included the right fusiform and parahippocampal gyrus (Figure 1.5). Finally, we found negative correlations between the duration of MDMA use and BM in three clusters: the first cluster included the left postcentral and inferior parietal gyrus; the second cluster included the right inferior frontal gyrus (pars triangularis) extending to the DLFPC; and the third cluster included the right superior temporal pole extending to the middle part of this region (Figure 1.6).

## Discussion

Our findings showed that the estimates of severity of use of heroin, alcohol, MDMA and cannabis were negatively associated with DLPFC and temporal cortex regional metabolism in polysubstance dependent individuals with prolonged abstinence from all these drugs. Alcohol use was further associated with lower metabolism in frontal premotor cortex and putamen. Furthermore, severity of stimulants use (cocaine and MDMA) was uniquely associated with inferior parietal/postcentral cortex metabolism. These associations were established after a prolonged period of abstinence, and after controlling for multiple confounding variables, thus surpassing the limitations of previous functional neuroimaging studies that were not specifically designed to study prolonged abstinence, and were confounded by important variables, such as withdrawal related symptoms or concurrent use of multiple substances. The associations found are in agreement with our initial predictions, and with the neuropsychological deficits typically aligned with the chronic use of these substances; these include reduced competency in executive functions (related to all classes of drugs), episodic memory (mainly observed on heroin, alcohol, MDMA and cannabis users), motor control (alcohol and cannabis users), and visuospatial attention (stimulants users) (see meta-analyses in [3], [8], [19], [20]).

As expected, the estimates of amount or duration of different classes of drugs were negatively associated with overlapping regions within the DLPFC (BA 9, 45, 46). This region has been linked with several of the key neuroadaptations associated with drug addiction, including drug conditioning, loss of self-control, and stimulus-driven compulsive behavior [2]. This finding is also in agreement with results from several neuropsychological studies showing cognitive deficits on working memory, planning or inhibition processes, which are associated with DLPFC functioning, across users of several drugs [3]. We also found negative associations between estimates of duration of heroin, alcohol, and MDMA use and regional metabolism on partially overlapping sections of the temporal cortex. These associates may substantiate the links between the intensity and duration of involvement with these drugs and the degree of memory attrition, which has been observed in longitudinal studies with users of these substances [21], [22]. It is worth mentioning that duration of heroin use was shorter to that of cocaine and alcohol use; however, previous brain structural findings indicate that duration of heroin use is a critical factor leading to brain damage, even in users with periods of use below five years [23]. Surprisingly, we failed to find significant associations between the severity of the substances used and overlapping limbic, striatal and cerebellar regions but this result could be explained from the fact that these regions play a greater role during current use and short-term abstinence [24]. In addition, we found relatively specific negative correlations between amount of cocaine use and regional metabolism in the inferior parietal cortex, and between alcohol duration and regions involved in motor programming and control (frontal precentral and putamen). The link between cocaine use and parietal dysfunction has been observed on previous functional imaging studies during cognitive challenges of attention and executive control [6], [25], [26]. The link between alcohol use and precentral and basal ganglia dysfunction is in agreement with structural imaging findings showing that function of these regions is significantly reduced in alcohol users compared to other forms of addiction [27]. Additional evidence comes from volumetric studies which associated volumetric measures of these regions and deficient motor skills in alcoholics [28]. These more specific findings could be used to design tailored interventions aimed to address specific aspects of frontal-subcortical executive functions in different profiles of substance abusers.

An important implication of this study is the fact that the link between severity of drug use and BM is still observable in substance abusers with prolonged periods of abstinence (an average of eight months in this sample). Therefore, quantity and duration of drug use may still be affect brain functioning after several months of successful cessation of drug use. This is particularly relevant in view of recent findings indicating that certain patterns of brain dysfunction can reliably predict mid-term and long-term relapse. Permanent brain alterations may also have important repercussions for the rehabilitation of SDI, since these alterations can affect the ability of SDI to assimilate the contents and participate in the activities of the rehabilitation programs [29], [30]. In fact, preliminary evidence suggests that cognitive rehabilitation techniques, such as errorless learning may be successful in compensating chronic cognitive deficits in substance abusers [31]. Thus, the brain networks impacted by severity of drug abuse can play a central role in clinical outcome and relapse and should become priority targets for therapeutic interventions.

Several possible limitations of our study should be considered and addressed by future research. Our findings were not obtained in “pure” users of each of the substances but in mostly polysubstance users. Although this limitation is inherent to the clinical literature on addiction (i.e., “pure” users of one single drug are quite rare among substance abusers enrolled in treatment programs), we attempted to control for the co-abuse of other drugs using statistics. A related confounding variable was the use of nicotine, which is ubiquitous among drug users but was not specifically assessed or controlled for. Furthermore, due to the use of a correlational design, this study cannot yield conclusions about cause-effect relationships between the use of drug and resting BM. However, given the agreement of our results with both resting state PET, activation PET/fMRI and addiction neuropsychology studies, we could hypothesize that, at least in part, the alterations observed in this population could be due to the quantity and duration related effects of the consumption of the substances under consideration in this study.

One last point to consider is that our findings could be due to premorbid brain alterations or the results of the interaction between the premorbid alterations and the neurotoxic effects of drug use. This question should be addressed through longitudinal designs. A related limitation is that no comparison with a healthy control database was performed. As we argued before, a healthy control group was not needed to test the main predictions of our study; however, the lack of controls has stopped us from making any assumption about the direction of these findings in relation to the healthy population.

## References

[1] Koob GF, Volkow ND (2010). Neurocircuitry of addiction.. Neuropsychopharmacology.

[2] Goldstein RZ, Volkow ND (2011). Dysfunction of the prefrontal cortex in addiction: neuroimaging findings and clinical implications.. Nat Rev Neurosci.

[3] Fernández-Serrano MJ, Pérez-García M, Verdejo-García A (2011). What are the specific vs. generalized effects of drugs of abuse on neuropsychological performance?. Neurosci Biobehav Rev.

[4] Aron JL, Paulus MP (2007). Location, location: using functional magnetic resonance imaging to pinpoint brain differences relevant to stimulant use.. Addiction.

[5] Lane SD, Steinberg JL, Ma L, Hasan KM, Kramer LA (2010). Diffusion tensor imaging and decision making in cocaine dependence.. PLoS One.

[6] Tomasi D, Goldstein RZ, Telang F, Maloney T, Alia-Klein N (2007). Widespread disruption in brain activation patterns to a working memory task during cocaine abstinence.. Brain Res.

[7] Gruber SA, Silveri MM, Yurgelun-Todd DA (2007). Neuropsychological consequences of opiate use.. Neuropsychol Rev.

[8] Kish SJ, Lerch J, Furukawa Y, Tong J, McCluskey T (2010). Decreased cerebral cortical serotonin transporter binding in ecstasy users: a positron emission tomography/[(11)C]DASB and structural brain imaging study.. Brain.

[9] de Win MM, Jager G, Booij J, Reneman L, Schilt T (2008). Neurotoxic effects of ecstasy on the thalamus.. Br J Psychiatry.

[10] King GR, Ernst T, Deng W, Stenger A, Gonzales RM (2011). Altered brain activation during visuomotor integration in chronic active cannabis users: relationship to cortisol levels.. J Neurosci.

[11] Pillay SS, Rogowska J, Kanayama G, Gruber S, Simpson N (2008). Cannabis and motor function: fMRI changes following 28 days of discontinuation.. Exp Clin Psychopharmacol.

[12] Martín-Santos R, Fagundo AB, Crippa JA, Atakan Z, Bhattacharyya S (2010). Neuroimaging in cannabis use: a systematic review of the literature.. Psychol Med.

[13] Goldstein RZ, Alia-Klein N, Tomasi D, Carrillo JH, Maloney T (2009). Anterior cingulate cortex hypoactivations to an emotionally salient task in cocaine addiction.. Proc Natl Acad Sci U S A.

[14] Zubieta JK, Heitzeg MM, Xu Y, Koeppe RA, Ni L (2005). Regional cerebral blood flow responses to smoking in tobacco smokers after overnight abstinence.. Am J Psychiatry.

[15] Verdejo-García AJ, López-Torrecillas F, Aguilar de Arcos F, Pérez-García M (2005). Differential effects of MDMA, cocaine, and cannabis use severity on distinctive components of the executive functions in polysubstance users: a multiple regression analysis.. Addict Behav.

[16] Boellaard R, O’Doherty MJ, Weber WA, Mottaghy FM, Lonsdale MN (2010). FDG PET and PET/CT: EANM procedure guidelines for tumour PET imaging: version 1.0.. Eur J Nucl Med Mol Imaging.

[17] Fehr C, Hohmann N, Gründer G, Dielentheis TF, Buchholz HG (2007). Tiagabine does not attenuate alcohol-induced activation of the human reward system.. Psychopharmacology (Berl).

[18] Volkow ND, Fowler JS, Wang GJ, Telang F, Logan J (2010). Cognitive control of drug craving inhibits brain reward regions in cocaine abusers.. Neuroimage.

[19] Jovanovski D, Erb S, Zakzanis KK (2005). Neurocognitive deficits in cocaine users: a quantitative review of the evidence.. J Clin Exp Neuropsychol.

[20] Zakzanis KK, Campbell Z, Jovanovski D (2007). The neuropsychology of ecstasy (MDMA) use: a quantitative review.. Hum Psychopharmacol.

[21] Hanson KL, Cummins K, Tapert SF, Brown SA (2011). Changes in neuropsychological functioning over 10 years following adolescent substance abuse treatment.. Psychol Addict Behav.

[22] de Sola S, Tarancón T, Peña-Casanova J, Espadaler JM, Langohr K (2008). Auditory event-related potentials (P3) and cognitive performance in recreational ecstasy polydrug users: evidence from a 12-month longitudinal study.. Psychopharmacology (Berl).

[23] Yuan Y, Zhu Z, Shi J, Zou Z, Yuan F (2009). Gray matter density negatively correlates with duration of heroin use in young lifetime heroin-dependent individuals.. Brain Cogn.

[24] Breiter HC, Rosen BR (1999). Functional magnetic resonance imaging of brain reward circuitry in the human.. Ann N Y Acad Sci.

[25] Barrós-Loscertales A, Bustamante JC, Ventura-Campos N, Llopis JJ, Parcet MA (2011). Lower activation in the right frontoparietal network during a counting Stroop task in a cocaine-dependent group.. Psychiatry Res.

[26] Bustamante JC, Barrós-Loscertales A, Ventura-Campos N, Sanjuán A, Llopis JJ (2011). Right parietal hypoactivation in a cocaine-dependent group during a verbal working memory task.. Brain Res.

[27] van Holst RJ, de Ruiter MB, van den Brink W, Veltman DJ, Goudriaan AE (2012). A voxel-based morphometry study comparing problem gamblers, alcohol abusers, and healthy controls. Drug Alcohol Depend.. In press.

[28] Sullivan EV, Deshmukh A, De Rosa E, Rosenbloom MJ, Pfefferbaum A (2005). Striatal and forebrain nuclei volumes: contribution to motor function and working memory deficits in alcoholism.. Biol Psychiatry.

[29] Johnson-Greene D, Adams KM, Gilman S, Junck L (2002). Relationship between neuropsychological and emotional functioning in severe chronic alcoholism.. Clin Neuropsychol.

[30] Zinn S, Stein R, Swartzwelder HS (2004). Executive functioning early in abstinence from alcohol.. Alcohol Clin Exp Res.

[31] Pitel AL, Perruchet P, Vabret F, Desgranges B, Eustache F (2009). The advantage of errorless learning for the acquisition of new concepts' labels in alcoholics.. Psychol Med.

[32] Tzourio-Mazoyer N, Landeau B, Papathanassiou D, Crivello F, Etard O (2002). Automated anatomical labeling of activations in SPM using a macroscopic anatomical parcellation of the MNI MRI single-subject brain.. Neuroimage.

